# Effect of the antenatal HIV testing scale up community intervention in South West, Nigeria: a cross-sectional study

**DOI:** 10.11604/pamj.2021.40.90.30230

**Published:** 2021-10-12

**Authors:** Akin Olufemi Oyebade, Monsur Akanmu Bello, Isola Oladimeji Familusi

**Affiliations:** 1 Osun State Agency for Control of AIDS, Osogbo, Nigeria; 2 Ministry of Health, Osun State, Nigeria

**Keywords:** Antenatal HIV testing, community intervention, cross-sectional study, health facility, HIV/AIDS, medical records, prevention of mother to child transmission, post-intervention, scale up, structural barriers

## Abstract

**Introduction:**

HIV/AIDS is a major killer of under five children, with about 25-30% of children born to infected mothers becoming infected with HIV in the absence of Prevention of Mother to Child Transmission of HIV (PMTCT) intervention. This study was carried out to assess the effectiveness of the Antenatal HIV Testing scale up community intervention to increase the uptake of Antenatal HIV Testing Testing. The intervention was implemented to increase the low coverages of Antenatal HIV Testing and PMTCT services in Osun State, South West, Nigeria.

**Methods:**

the cross-sectional design was utilized for the study. Data was collected using data collection instruments administered to 600 respondents from the 30 Local Government Areas (LGAs) of the State. Data was also extracted from Medical Records generated from the Health Facilities where pregnant women received HIV Testing.

**Results:**

the study revealed that 72% of adult respondent had adequate knowledge of the importance of Antenatal HIV Testing while 98% of female respondent had accepting attitude to getting tested for HIV during their next pregnancy. The number of pregnant women who received HIV Testing increased from 6,254, pre-intervention (April to August 2019) to 8,240, post-intervention (September 2019-January 2021).

**Conclusion:**

the Antenatal HIV Testing scale up community intervention is effective in increasing the utilization of Antenatal HIV Testing by increasing awareness and attitude to HIV testing in the community. Thus, efforts to scale it up in Nigeria should be accelerated to improve PMTCT services and reduce Mother to Child Transmission of HIV.

## Introduction

In Nigeria, it is estimated that almost one million women and children die yearly, largely from preventable causes. Nigeria is the second largest contributor to under-five mortality globally, accounting for 13% of all under-five deaths. HIV/AIDS is a major killer of under five children, especially in developing countries [1]. About 25-30% of children born to infected mothers without intervention become infected with HIV and almost all of them die before 5 years of age. Increasing the proportion of pregnant women who receive HIV counselling and testing during antenatal care will contribute to improving the coverage of PMTCT services which in turn will assist to reducing or eliminating mother-to-child transmission of HIV (MTCT) [2]. In Osun State, under-five mortality is on the increase, from 56 per 1,000 [3] in 2011 to 101 per 1,000 in 2017 [4]. The proportion of pregnant women who received HIV testing and counseling during antenatal care decreased from 41.6% [3] in 2011 to 39.8% [4] in 2017. PMTCT Services coverage declined from 31.6% [5] in 2011 to 17.6% [6] in 2017, while the mother-to-child transmission of HIV (MTCT) rate increased from 32% [5] to 35% [6] during this period. One of the responses by Government to the challenges described above is the Saving One Million Lives (SOML) Program. SOML is meant to improve maternal and child health outcomes and has been implemented in Osun State to improve maternal, newborn and child health, treatment of important childhood diseases, child nutrition, immunization, malaria control and elimination of mother to child transmission (eMTCT) of HIV services [7].

The Antenatal HIV Testing scale up community intervention was implemented to eliminate mother to child transmission of HIV by increasing antenatal HIV Testing and generating demand for PMTCT Services. The intervention engaged Community Mobilizers to link pregnant women to Antenatal Services and Antenatal HIV Testing through advocacy building, awareness creation and conditional cash transfer. The community approach led to increased enrolment of pregnant women into antenatal care and increased uptake of HIV Testing Services at health facilities in the state. At the end of the intervention 15,681 pregnant women were successfully linked to accredited health facilities to access antenatal care and HIV Testing Services between April 2019 to January 2020. In order to eliminate MTCT, at least 95% of pregnant women should receive HIV counseling and testing during antenatal care and at least 95% PMTCT coverage is required [2]. Eliminating mother to child transmission of HIV will contribute significantly to the achievement of SOML outcome: to reduce under-five mortality [7].

This study was conducted to determine the effectiveness of the Antenatal HIV Testing Community Intervention to increase utilization of HIV Testing among Pregnant women in Osun State. The result of the study is expected to provide justification for adopting and expanding the intervention. The need to assess the effectiveness of the intervention is particularly important in the face of the low uptakes of Antenatal HIV Testing and PMTCT services, despite significant progress in supply of Antenatal HIV Testing and PMTCT services. Innovative interventions that address demand side barriers are therefore required by HIV programmers if they must increase uptake of Antenatal HIV Testing Services significantly. This will also allow for improvement of PMTCT coverage, reduce MTCT and consequently better Maternal and Child Health outcomes [3-6].

The study will test the following hypotheses: 1) the antenatal HIV testing scale up community intervention increased knowledge of people about the importance of Antenatal HIV Testing. 2) The antenatal HIV testing scale up community intervention increased knowledge of people about the barriers to utilization of Antenatal HIV Testing. 3) The Antenatal HIV Testing scale up community intervention improved attitude of women to utilization of Antenatal HIV Testing. 4) The Antenatal HIV Testing testing scale up community intervention increased utilization of Antenatal HIV Testing services in the Health Facilities.

The aim of the study is: 1) to estimate the effect of the Antenatal HIV Testing scale up community intervention on uptake of Antenatal HIV Testing Services (HTS) in Osun State. The objectives of the study are: 1) to determine proportion of people with adequate knowledge of the importance of Antenatal HIV Testing; 2) to determine proportion of people with adequate knowledge of the barriers to utilization of Antenatal HIV Testing; 3) to determine proportion of women with right attitude to utilizing Antenatal HIV Testing; 4) to compare utilization of Antenatal HIV Testing Services, pre and post intervention.

## Methods

**Research design:** this study utilized the cross-sectional design to assess the effect of the Antenatal HIV Scale Up Community Intervention on uptake of Antenatal HIV Testing in Osun State.

**Study participants:** the study participants were adult males and females and women of reproductive age-group residing in the 30 LGAs of Osun State, Nigeria.

### Study setting

The study was carried out in Osun State, an inland state in South West, Nigeria with capital city at Osogbo. Osun State, created from the old Oyo State on the 27^th^ of August, 1991, covers a total landmass of about 9,251 square kilometers and is located within latitude 6.55° and 8.10° North and longitude 3.55° and 5.05° East and is bounded by Ogun State to the South, Kwara State to the North, Oyo State to the West and Ekiti and Ondo State to the East. It is divided into three federal senatorial districts, each of which is composed of two administrative zones. It consists of thirty Local Government Areas (LGAs) and Area Office and there are over 200 major towns and several villages in the state. Most of the area of the State is situated under tropical rain forest vegetation with patches of savannah in the Northern part and minerals resources found include gold and kaolin. It is also blessed with presence of many rivers and streams which serves the water needs of the state with the state's name derived from the Osun River. The people of Osun are predominantly Yorubas and the language is Yoruba, although there are variations in intonation and accent in and across the towns and cities. The major sub-ethnic groups are Ife, Ijesha, Oyo and Igbomina of the Yoruba people, although there are also people from other parts of Nigeria. Yoruba and English are the official languages. The inhabitants are mostly farmers, producing such food crops as yam, maize, cassava, beans and cocoyam with cash crops grown including tobacco and palm produce but are also culturally rich in all spheres of life such as arts, literature, music and other social activities in the state [8]. Osun State has a projected 2016 population of 4,705,600 according to the National Bureau of Statistics [9].

### Method of development and validation of data collection instrument

The data collection instruments used comprised of two questionnaires (one for data collection from community adult respondents and the other from females of reproductive age group) and one data extraction tool for collection of data from health facilities medical records. The data collection instruments were developed by the authors after careful review of literature, followed by development of indicators required for hypotheses testing. Semi-structured, closed and open-ended and interviewer administered questionnaires and data collection tool with specified indicators were finally designed. The adult´s respondent questionnaire contained two sections and nine questions. Section 1 collected data on socio-demographic characteristics while Section 2 collected data on respondent knowledge of Antenatal HIV Testing. The female of reproductive age questionnaire contained two sections and eight questions. Section 1 collected data on socio-demographic characteristics while Section 2 collected data on attitude and practice of female respondent with respect to Antenatal HIV Testing. The Data Extraction Tool contained two sections. Section 1 collected data on characteristics of the health facilities while Section 2 was used to extract data on the number of pregnant women that received Antenatal HIV Testing pre and post intervention from medical records.

We pre-tested the data collection instrument on a sample of 20 respondents in five communities to validate it. Pretesting helped to detect deficiencies and ambiguities in the questionnaires and data extraction tool. Appropriate corrections were made to the questionnaires and data extraction tools after pretesting by eliminating irregular questions and questions having same interpretation and ensuring utilization of SMART indicators. This lessened the time taken to complete the questionnaire and data collection tool. The initial drafts had overall 26 questions that were reduced to 17 questions. Interviewers were also trained on providing guidance to respondents on interpretation of questionnaire items and proper transcription of data to increase validity.

**Procedure for data collection:** data was collected using data collection instruments administered to 600 respondents (300 Adult Respondents and 300 Women of Reproductive Age) from the 30 LGAs of the State. Data was also extracted from Medical Records obtained from 30 selected health facilities where pregnant women were linked to access Antenatal Care and Antenatal HIV Testing.

**Source of data:** data was sourced from 600 respondents and medical records on Antenatal Attendance from 30 Health Facilities utilizing data collection instrument (questionnaire and data extraction tool).

**Variable:** the study had the following variables: 1) predictor variables comprise of age, sex, occupation, type of facility; 2) exposure variable prior utilization of Antenatal HIV Testing; 2) outcome variable-knowledge of importance of Antenatal HIV Testing, knowledge of barriers to antenatal HIV testing, attitude to Antenatal HIV Testing, antenatal HIV testing attendance.

**Sampling method:** convenient sampling method was utilized for the study. The 600 respondents were divided into 300 Adult Respondents and 300 women of reproductive age. The 300 Adult Respondents were selected at 10 per LGA while the 300 women of reproductive age were selected at 10 per LGA respectively. The 30 Health Facilities where data was collected were selected based on higher monthly attendance. The non-usage of random sampling introduces potential source of bias especially selection bias.

**Sample size:** the sample size was estimated using the Cochran formula for cross-sectional studies below.


$$n=\frac{z^{2}\times p\times q}{d^{2}}$$

Where n = sample size: z = level of significance; p= prevalence of attribute in the population; Assume p = 40% = 0.4 (most recent Antenatal HIV Testing coverage = 39.8%); q = 1-p =1-0.4= 0.6; d= precision. Setting precision at 4% then d=4%=0.04 z=1.96. Therefore


$$\begin{array}{ll} n & =\frac{1.96^{2}\times 0.6\times 0.4}{0.04^{2}}\\ n & =\frac{3.8416\times 0.24}{0.04^{2}}=\frac{0.922}{0.0016}\\ n & =600 \end{array}=576$$

### Method of data analysis

Epi Info Build 7.2.4 and Microsoft Excel Version 2019 were utilized to determine the following: 1) Simple descriptive statistics to characterize the respondents; 2) frequency distribution of level of knowledge in relation to Antenatal HIV testing services; 3) frequency distribution of attitude to utilization of Antenatal HIV testing services; 4) association between accepting attitude to utilization of antenatal HIV testing services and prior utilization of Antenatal HIV Testing using Chi Squared test approximated by Fischer Exact Test; 5) utilization rate of Antenatal HIV Testing pre and post intervention.

**Ethical consideration:** ethical approval for this study was obtained from Ethical Review Board of the Osun State Agency for Control of AIDS.

## Results

Adult Respondents comprised of Adult Males and Females with mean age of 52 years. Adult female respondents formed 62% with mean age of 49 years while adult male respondents formed 38% with mean age of 55 years. Fourty-two (42%) of Adult Respondents were skilled workers while 58% were engaged in trade (Table 1). Female respondents of reproductive age had mean age of 32 years with 42% as skilled workers while 58% were engaged in trade (Table 1). Ninety-nine percent (99%) of health facilities where pregnant women were linked were public health facilities while only 1% was private health facility. Ninety-seven percent (97%) of Health Facilities were Primary Health Centres (PHCs) while 3% were secondary health facilities (Table 1). Seventy-two percent (72%) of community respondent had adequate knowledge of the importance of Antenatal HIV Testing (to enable pregnant women determine their HIV status, detect pregnant women who are HIV positive, ensure HIV positive pregnant women are referred for PMTCT and avoid having babies that are born HIV positive). Twenty-five (25%) of Adult Respondents had adequate knowledge of the barriers to uptake of Antenatal HIV Testing (lack of transportation fare, lack of support from husband, lack of information and poor attitude of health worker) however, 29% believed that lack of transportation fare as the major barrier to uptake of Antenatal HIV Testing (Table 2).

**Table 1:** characteristics of respondents and health facilities

Distribution of adult respondents						
**Sex**	**Frequency**	**Percentage**	**Mean Age**	**Occupation**	**Frequency**	**Percentage**
Female	185	62%	49 years	Skilled	126	42%
Male	115	38%	55 years	Trade	174	58%
All	300	100%	52 years	All	300	100%
Distribution of females respondents of reproductive age						
**Sex**	**Frequency**	**Percentage**	**Mean Age**	**Occupation**	**Frequency**	**Percentage**
Female	300	100%	32 years	Skilled	123	41%
All	300	100%		Trade	177	59%
-	-	-		All	300	100%
Distribution of health facilities by type & level of care						
**Type**	**Frequency**	**Percentage**	**Level of Care**	**Frequency**	**Percentage**	-
Private	4	1.1%	PHC	354	97.25%	-
Public	360	98.9%	SHC	10	2.75%	-
All	364	100%	TH	0	0	-
-	-	-	Total	364	100%	-

**Table 2:** knowledge and attitude of female respondents in relation to antenatal HIV testing services

Proportion of Adult Respondents with adequate knowledge of importance of Antenatal HIV testing	Frequency	Percent
To enable pregnant-women determine their HIV status	44	14.72%
To detect pregnant women who are HIV positive	20	6.69%
To ensure HIV positive pregnant women are referred for PMTCT	8	2.68%
To avoid having babies that are born HIV positive	13	4.35%
All options	215	71.56%
Total	300	100%
**Proportion of Adult Respondents with adequate knowledge of the barriers to uptake of Antenatal HIV Testing**	**Frequency**	**Percent**
Lack of transportation fare	87	29.19%
Lack of support from husband	27	9.06%
Lack of information about Antenatal HIV Testing	21	7.05%
Poor attitude of health worker	8	2.68%
None of the above	82	27.52%
All the options	75	24.5%
Total	300	100%
**Proportion of female respondents of reproductive age with right attitude towards accessing antenatal HIV testing**	**Frequency**	**Percent**
The desire to know HIV status	46	15.75%
The desire to ensure baby is born HIV negative	33	11.30%
The possibility of receiving cash incentive	23	7.88%
The availability of Antenatal services	5	1.72%
All options	193	63.35%
Total	300	100%

**Figure 1:**
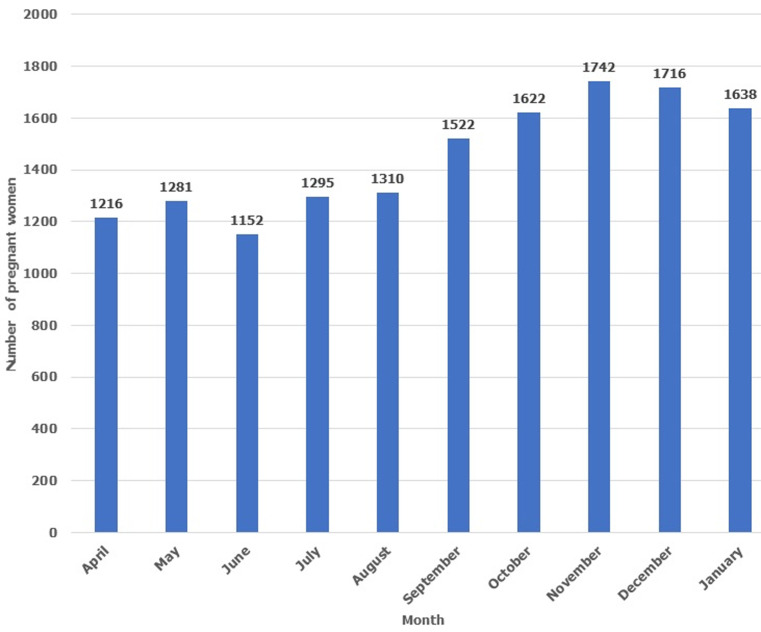
number of pregnant women who accessed antenatal HIV testing (April 2019-January 2020)

**Figure 2:**
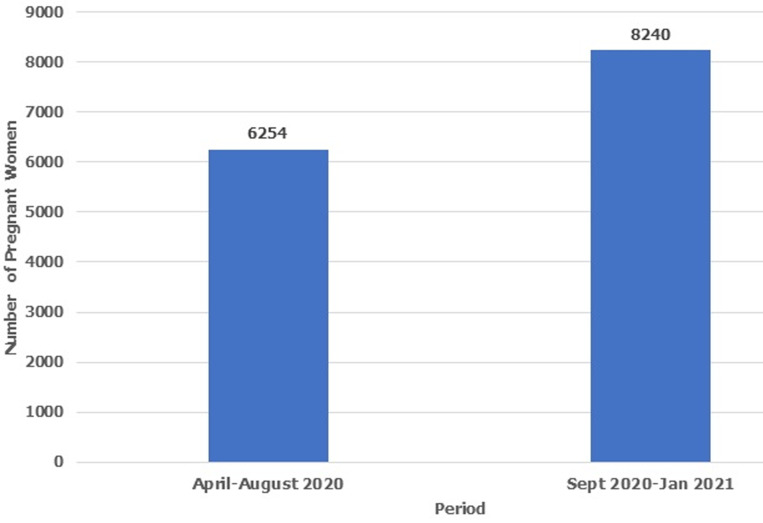
number of pregnant women who accessed antenatal HIV testing (pre and post intervention)

Sixty-three percent (63%) of female respondent had right attitude to getting tested for HIV during next pregnancy (desire to know HIV status, desire to ensure baby is born HIV negative, and availability of Antenatal services) and the possibility of receiving cash incentive (Table 2). Ninety-two percent (92%) of female respondent attended Antenatal Clinic and received HIV Testing during their last pregnancy while 98% of female respondent planned to attended Antenatal Clinic and get tested for HIV during next pregnancy (Table 3). Odds ratio of association between accessing Antenatal HIV Testing during last pregnancy and plan to access Antenatal HIV Testing for her next pregnancy was 5.96 at p-value of 0.234 (Table 3). The total number of pregnant women who accessed Antenatal HIV Testing monthly increased from pre-intervention levels in April 2019 - 1, 216, May 2019 - 1,281, June 2019 - 1,152, July 2019 - 1,295 and August 2019 - 1,310 to higher post intervention levels-September 2020-1, 522, October 2020-1,622, November 2020-1, 742, December 2019-1, 716 and January 2021-1, 638 (Figure 1). The total number of pregnant women who attended Antenatal HIV Testing increased by 32% from 6,254 Pre-Intervention-April-August 2019 to 8,240 Post Intervention-Sep 2019-Jan 2020 (Figure 2).

**Table 3:** accepting attitude to utilization of antenatal HIV testing services

Accepting Attitude to utilization of Antenatal HIV Testing (Prior Pregnancy)		Frequency	Percent	
Proportion of female respondents who accessed antenatal HIV testing during last pregnancy		275	91.6%	
Total		300	100%	
**Accepting Attitude to utilization of Antenatal HIV Testing (Future Pregnancy)**		**Frequency**	**Percent**	
Proportion of Female Respondents who accessed Antenatal HIV testing during last pregnancy		297	99%	
Total		300	100%	
**Association between utilization of Antenatal HIV Testing last pregnancy and Plan to access Antenatal HIV Testing next pregnancy**				
Chi Squared Test approximated by Fischer Exact Test Odds Ratio=5.96 P-Value=0.2304		Plan to utilize Antenatal HIV Testing next pregnancy		
		**Yes**	**No**	**Total**
Utilized Antenatal HIV Testing previous pregnancy	**Yes**	273	2	275
	**No**	24	1	25
	**Total**	297	3	300
Proportion of women who had ANC HTS last pregnancy= (275 x 100)/300 = 92%				
Proportion of women who plan to access ANC HTS for next pregnancy= (297 x 100)/300 = 92%				
Odds of Plan for ANC HTS future pregnancy among women with prior ANC HTS = 273=136.5 2				
Odds of Plan for ANC HTS future pregnancy among women without prior ANC HTS= 24=241				
Odds Ratio=(136.5)/24=5.96				

## Discussion

The study showed a high knowledge (72%) of the importance of antenatal HIV testing among adult respondents. This means the intervention was effective in increasing awareness about the importance of Antenatal HIV Testing in the community. This confirms our first hypothesis and is supported by the study by Gazimbi *et al*. [10] which showed that high level of HIV awareness was associated with high uptake of Antenatal HIV Testing. An increase in Antenatal HIV Testing from 26.7% to 59.5%, p-value <0.001 over time was detected in their study looking at effect of enabling factors on Antenatal HIV testing. Our finding is also supported by the study by Hạnh *et al*. [11] which showed that structural factors associated with lack of Antenatal HIV test included not being informed about Antenatal HIV Testing and PMTCT at first antenatal care visit (AOR 7.4, 95% CI 2.6-21.1).

An unexpectedly low proportion (25%) of adult respondents were found to have adequate knowledge of barriers to uptake of Antenatal HIV Testing. This means the intervention was not able to adequately increase awareness of the barriers to utilizing Antenatal HIV Testing. However, 29% of adult respondents believed that lack of transportation was a major barrier. This is consistent with the study by Fagbamigbe *et al*. [12] which showed that lack of transport fare constituted about 17% of all barriers to accessing antenatal care a high proportion (92%) of female respondent claimed to have utilized Antenatal HIV Testing during their previous pregnancy. This reported high Antenatal HIV Testing coverage is inconsistent with results from previous MICS [3,4]. Which puts Antenatal HIV Testing coverage at 41.6% and 39.8% for 2011 and 2017 respectively. The result obtained in the study could be due to low sample size, selection bias or it may mean the Antenatal HIV Testing coverage has increased over the years. This therefore requires further studies of larger sample sizes or a survey to clarify. A higher proportion of female respondent (98%) plan to access Antenatal HIV Testing in their next pregnancy, which infers that the intervention is effective in increasing accepting attitude of women to accessing Antenatal HIV Testing.

The odds of a woman planning to access Antenatal HIV Testing for her next pregnancy was 6 times higher among women who accessed Antenatal HIV Testing during last pregnancy compared to those who did not access Antenatal HIV Testing during last pregnancy, although not statistically significant (p-value 0.234). This means that women who access Antenatal HIV Testing previously are more likely to continue using Antenatal HIV Testing when provided with the right information. This is supported by the study by Gebeyehu *et al*. [13]. which revealed that the odds of acceptance of Antenatal HIV Testing was higher among respondents who had awareness about MTCT (AOR = 2.602, 95%; CI: 1.408-4.809) than their counterparts. It was also higher among respondents who had perception of the benefit of Antenatal HIV Testing (AOR = 1.838, 95%; CI: 1.089-3.104) than those who did not. Participants who were knowledgeable about the PMTCT were also more likely to accept testing (AOR = 1.715, 95%; CI: 1.030-2.855) than their counterparts.

A review of the utilization pattern of antenatal HIV Testing services by pregnant women showed a steady increase over a 10-month period that covers the pre and post intervention period. However, the utilization pattern in the post intervention period were higher comparing the individual months (Figure 1). Medical records also showed an increase in the total number of pregnant women who received Antenatal HIV Testing from 6,254 (April-August 2019) to 8,240 (September 2019-Jan 2020), an increase of 32% (Figure 2). This means that the Antenatal HIV Testing testing scale up community intervention was effective in increasing utilization of Antenatal HIV Testing in confirmation of our fourth hypothesis. This finding is consistent with the result of the study by Madhivanan *et al*. [14] which revealed that community mobilization improved uptake of antenatal care and Antenatal HIV testing services. The study showed that 43.3% of pregnant women in a mobile Antenatal HIV Testing intervention compared to 72.5% in a community mobilization plus mobile Antenatal HIV Testing intervention received antenatal care (< 0.001) while 97% of pregnant women in the mobile Antenatal HIV Testing intervention compared to 98.6% of pregnant women in the community mobilization + mobile Antenatal HIV Testing intervention consented to HIV testing (< 0.001).

The limitation to this study is the lack of a baseline study or pre-intervention study which would have made comparison with post-intervention results more accurate. The sample size however is a strength providing adequate power for the study. Another limitation of the study is that although the study participants are not significantly different from adults and female of reproductive health population in other location in terms of the exposure variables, however the result cannot be safely generalized to adults and female of reproductive health population in other locations because of utilization of convenient sampling method. Future research would need to be designed with both pre-intervention and post-intervention phases to enable accurate comparison. There would also be the need to utilize a probability sampling method to permit generalization of the study result to the target population. Also, there would be need to improve the design of the Antenatal HIV Testing scale up community intervention to enable it address the low level of knowledge of the barriers to utilization of Antenatal HIV Testing.

## Conclusion

Our study shows that the Antenatal HIV Testing scale up community intervention was effective in increasing the utilization of Antenatal HIV Testing in Osun State. This means that the Community Mobilization (Community Awareness, Education and Empowerment) approach helped to mobilize more pregnant women for Antenatal HIV Testing at the health facilities. It resulted in increased knowledge of importance of Antenatal HIV Testing, right attitude to accessing Antenatal HIV Testing and accepting attitude to utilization of Antenatal HIV Testing leading to more pregnant women getting tested for HIV at the health facilities. We therefore make recommendation for the adoption of the Antenatal HIV Testing scale up community intervention by HIV programmers. The intervention will assist programmers to increase the coverage of Antenatal HIV Testing, generate demand for PMTCT services and contribute to elimination of Mother to Child Transmission of HIV. This is important, especially as all hands are on the deck to ensure achievement of the Joint United Nations Programme on HIV/AIDS (UNAIDS) goal and Sustainable Development Goal (SDG) to end AIDS by 2030. It is also recommended that poverty reduction programmes be scaled up to help remove the financial barriers to accessing Antenatal Care and Antenatal HIV Testing Services in the State

### What is known about this topic

- It is already known through previous studies that high level of HIV awareness is associated with high uptake of Antenatal HIV Testing [10] and that Community Mobilization enhances mobile Antenatal HIV Testing.

### What this study adds

- Our study adds the knowledge that Community mobilization increases Antenatal HIV Testing at the health facilities by mobilizing pregnant women from the community to the health facilities for HIV testing;
- It also revealed a positive association between accessing Antenatal HIV Testing during last pregnancy and plan to access Antenatal HIV Testing for next pregnancy.
